# Nucleocytoplasmic transport of nucleocapsid proteins of enveloped RNA viruses

**DOI:** 10.3389/fmicb.2015.00553

**Published:** 2015-06-02

**Authors:** Wahyu N. Wulan, Deborah Heydet, Erin J. Walker, Michelle E. Gahan, Reena Ghildyal

**Affiliations:** ^1^Centre for Research in Therapeutic Solutions, University of Canberra, Bruce, ACTAustralia; ^2^Faculty of Education, Science, Technology and Mathematics, University of Canberra, Bruce, ACTAustralia

**Keywords:** enveloped RNA viruses, nucleocapsid protein, nuclear localization, nucleolar localization, antiviral responses

## Abstract

Most viruses with non-segmented single stranded RNA genomes complete their life cycle in the cytoplasm of infected cells. However, despite undergoing replication in the cytoplasm, the structural proteins of some of these RNA viruses localize to the nucleus at specific times in the virus life cycle, primarily early in infection. Limited evidence suggests that this enhances successful viral replication by interfering with or inhibiting the host antiviral response. Nucleocapsid proteins of RNA viruses have a well-established, essential cytoplasmic role in virus replication and assembly. Intriguingly, nucleocapsid proteins of some RNA viruses also localize to the nucleus/nucleolus of infected cells. Their nuclear function is less well understood although significant advances have been made in recent years. This review will focus on the nucleocapsid protein of cytoplasmic enveloped RNA viruses, including their localization to the nucleus/nucleolus and function therein. A greater understanding of the nuclear localization of nucleocapsid proteins has the potential to enhance therapeutic strategies as it can be a target for the development of live-attenuated vaccines or antiviral drugs.

## Introduction

The nucleocapsid protein of RNA viruses is essential for virus assembly, encapsidating the genomic RNA in preparation for packaging in the virion (Hunter, 2007). In positive strand RNA viruses (PSVs), the capsid or nucleocapsid protein is the major, often the only structural protein. In negative strand RNA viruses (NSVs), the nucleocapsid protein has an essential role in viral replication and transcription (Whelan, 2013) in addition to its role in assembly. For most enveloped RNA viruses, both replication and assembly occur in the cytoplasm (with the exception of orthomyxovirus, bornavirus, retroviruses, and hepatitis delta virus whose replication occurs in the nucleus; Whelan, 2013). Interestingly, the nucleocapsid protein (variously named N, NP, NC, or C protein) of many cytoplasmic RNA viruses (PSVs and NSVs) transiently localizes to the nucleus and/or nucleolus. There is limited information about the nuclear function of cytoplasmic RNA virus nucleocapsid proteins, but evidence suggests supporting roles in successful viral replication by interfering with or inhibiting the host antiviral response (Ahmed and Lyles, 1998; Ahmed et al., 2003; Lee et al., 2006; Takayama et al., 2012) as has been described for several RNA virus structural proteins.

The nucleocapsid protein of mouse hepatitis virus (MHV, *Coronaviridae*) and infectious bronchitis virus (IBV, *Coronaviridae*) localizes to the nucleolus of infected cells where it may regulate/delay cell cycle progression (Wurm et al., 2001), while nuclear localization of vesicular stomatitis virus (VSV, *Rhabdoviridae*) M, rabies (*Rhabdoviridae*) P, and Measles virus (MV, *Paramyxoviridae*) N protein (Ahmed et al., 2003; Schnell et al., 2010; Takayama et al., 2012) is associated with the pathogenesis of viral infection. Nuclear localization of the nucleocapsid protein of porcine respiratory and reproductive syndrome virus (PRRSV, *Arteriviridae*) appears to be essential for optimal virus replication and inhibition of cellular antiviral processes (Lee et al., 2006); in an analogous fashion, the successful production of infectious progeny of respiratory syncytial virus (RSV, *Paramyxoviridae*) and Nipah virus (*Paramyxoviridae*) requires nuclear localization of the matrix (M) protein (Ghildyal et al., 2005, 2009; Wang et al., 2010).

The aim of this review is to summarize current literature on the role of nuclear/nucleolar localization of nucleocapsid proteins of cytoplasmic PSVs and NSVs, which may lead to similar investigations in other related virus families. Most of our knowledge in this area derives from studies of viruses belonging to the *Coronaviridae, Arteriviridae, Flaviviridae*, and *Paramyxoviridae* families and these will be the main focus (**Table 1**), with reference to other virus families when required. *Coronaviridae* and *Arteriviridae* are classified together into the order *Nidovirales*; for the sake of simplicity and due to the close relationship among these viruses, both families will be discussed together.

**Table 1:** Classification of enveloped RNA viruses discussed in the review.

Family	Sub-family	Genus	Species
*Flaviviridae*		Flavivirus	Dengue virus, Japanese Encephalitis virus (JEV), West Nile virus
		Hepacivirus	Hepatitis C virus
*Coronaviridae*	*Coronavirinae*	Alphacoronavirus	Transmissible gastroenteritis virus (TGEV)
		Betacoronavirus	Severe acute respiratory syndrome coronavirus (SARS-CoV), mouse hepatitis virus
		Gammacoronavirus	Infectious bronchitis virus
*Arteriviridae*		Arterivirus	Lactose dehydrogenase elevating virus, Equine arterivirus, Porcine respiratory and reproductive syndrome virus
*Paramyxoviridae*	*Paramyxovirinae*	Morbillivirus	Measles virus, Canine distemper virus (CDV), Rinderpest virus
		Henipavirus	Nipah virus
		Respirovirus	Sendai virus
	*Pneumovirinae*	Pneumovirus	Respiratory syncytial virus

**FIGURE 1:**
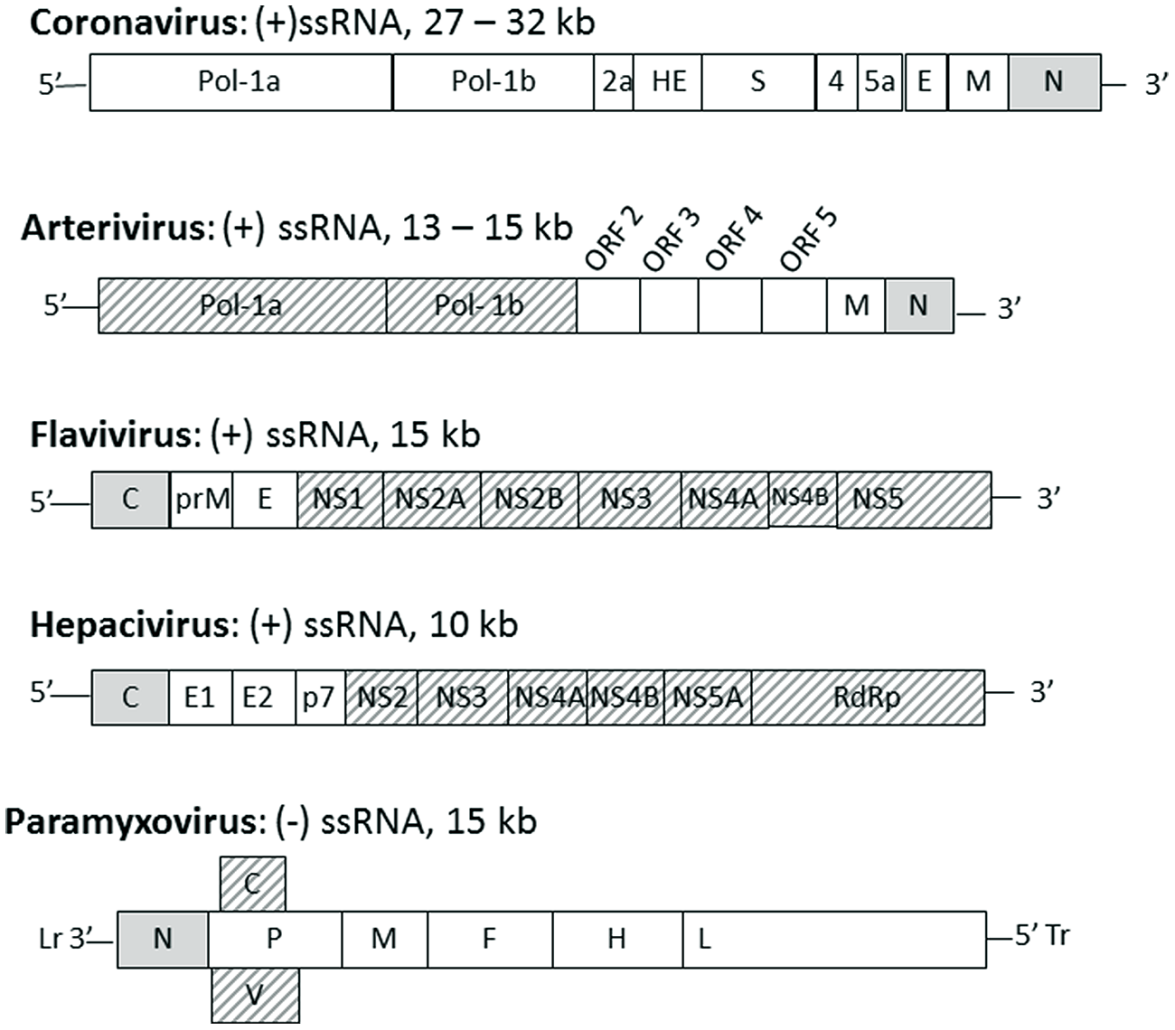
**The position of nucleocapsid protein in the genome of selected enveloped RNA virus families**. Genomes of the named virus families are indicated schematically with each gene indicated by a box with family name, genome type and length indicated above each schematic. The gene for nucleocapsid protein (N or C) is indicated by a gray box, other structural proteins are indicated by white boxes and non-structural proteins are indicated by hashed boxes. Lr, leader; Tr, trailer.

The location of the nucleocapsid gene within the genome of selected viruses from the named families is shown in **Figure 1**. The nucleocapsid [N, NC, or C (core or capsid)] gene is in the first position (3′ end) in Paramyxovirus genomes and (5′ end) in Flavivirus and Hepacivirus genomes. The nucleocapsid gene of Coronavirus and Arterivirus is in the last position (3′ end) of these genomes, but the first gene to be copied into a negative sense subgenomic mRNA template for mRNA transcription.

## The Cytoplasmic Role of RNA Virus Nucleocapsid Protein

The nucleocapsid protein of RNA viruses is a structural protein found in a complex with the genomic RNA. The role of genome encapsidation varies across PSVs and NSVs, but usually comprises one or more of the following functions; [1] to protect the genome from cellular nucleases, [2] to form the ribonucleoprotein transcriptase complex, and [3] to package the genome into new infectious particles prior to budding.

### Nucleocapsid Protein of NSVs

The NSV nucleocapsid protein protects genomic viral RNA against ribonuclease degradation and high salt concentration, as well as maintaining the rigidity of the RNA structure following cycles of folding/dissociation/refolding during the replication cycle (Lynch and Kolakofsky, 1978; Mir and Panganiban, 2006; Raymond et al., 2010). The nucleocapsid protein is the driving force for the formation of ribonucleoprotein (RNP) complexes that are the replicative/transcriptive unit, as well as for assembly and packaging into the virion. These functions are facilitated through its propensity to self-associate and ability to interact with other RNP components, including RNA and viral polymerase subunits (Becker et al., 1998; DiCarlo et al., 2007; Green et al., 2011; Sun et al., 2012). Nucleocapsid protein has an inherent ability to interact with the phosphoprotein (P; polymerase subunit and cofactor). This interaction is particularly important because association between nucleocapsid and RNA is not specific; instead, specificity is conferred via the nucleocapsid-phosphoprotein interaction, such that RNA associated with the nucleocapsid-phosphoprotein complex is the form recognized by RNA polymerase (Myers et al., 1999; Longhi, 2009). Additionally, the NSV phosphoprotein acts as a chaperone for the nucleocapsid protein restricting non-specific encapsidation of non-viral RNA (Curran et al., 1995; Errington and Emmerson, 1997). The nucleocapsid protein of VSV, in association with the P protein, is required for genome encapsidation and transcription (Zhang et al., 2008; Morin and Whelan, 2014) with mutations in its C-terminus resulting in reduction of viral particle production in cell culture (Heinrich et al., 2012). In RSV and Sendai virus (*Paramyxoviridae*), it is the N-terminal of the nucleocapsid protein that plays a similar role in genome encapsidation (Buchholz et al., 1993; Myers and Moyer, 1997; Khattar et al., 2000).

### The Nucleocapsid/Capsid/Core Protein of PSVs

Similar to the NSV nucleocapsid protein, the nucleocapsid/capsid/core protein of PSVs binds tightly to the genomic RNA, drives RNA packaging and sometimes, serves as a nucleic acid chaperone. Flavivirus (*Flaviviridae*) C protein plays an important role during assembly and budding of infectious particles. Mutations in C protein lead to the inhibition of infectious particle release while causing increased production of defective particles (Jones et al., 2011). The hepatitis C virus (HCV, *Flaviviridae*) core protein and the N protein of coronaviruses severe acute respiratory syndrome coronavirus (SARS-CoV) and transmissible gastroenteritis virus (TGEV) are potent RNA chaperones that can resolve RNA misfolding and promote annealing of complementary sequences (Rajkowitsch et al., 2007; Zuniga et al., 2007; Sharma et al., 2010). Most of the evidence for chaperone activity is derived from *in vitro* investigations and although these proteins clearly have the ability to function as RNA chaperones, the importance of this function in infection is not clear.

## Non-Encapsidation Roles of Nucleocapsid Proteins

The N/NC/C proteins of both NSVs and PSVs have functions in addition to their RNA stabilizing, encapsidating and, in the case of NSVs, viral transcriptase activities. The flavivirus core protein interacts with several host cell proteins and is implicated in disease development through its roles in apoptotic pathways as well as regulation of the innate immune response (Urbanowski et al., 2008). Similarly, the coronavirus N protein interferes with cellular antiviral responses (McBride et al., 2014). The N protein of morbilliviruses (*Paramyxoviridae*) has been shown to regulate interferon signaling via interference with the signal transducer and activator of transcription (STAT) signaling pathway (Takayama et al., 2012). Increasing literature suggests that at least some of the non-encapsidating functions of the nucleocapsid proteins of NSVs and PSVs derive from their ability to localize to the nucleus or nucleolus of the infected cells.

## Nuclear Localization of Nucleocapsid Proteins of RNA Viruses

Despite undergoing replication in the cytoplasm, the nucleocapsid protein of several NSVs and PSVs localizes in the nucleus or nucleolus of infected cells during infection. This nuclear localization usually takes place early in the infectious cycle as soon as the nucleocapsid protein is translated, possibly to perform non-structural functions, followed by return to the cytoplasm in the late stage of infection to participate in assembly (Tijms et al., 2002; Yoo et al., 2003; Sato et al., 2006). Although some progress has been made in our understanding of the nucleocytoplasmic shuttling of several nucleocapsid proteins, thus far, no definitive single molecule studies have been undertaken; such studies will be important to elucidate the finer details of nuclear transport pathways in the future.

### Nucleocytoplasmic Transport

Eukaryotic cells sequester their genome in the nucleus, which is surrounded by the double lipid bilayer structure of the nuclear envelope (NE). The only avenue for transport into and out of the nucleus is via the NE-embedded nuclear pore complexes (NPCs) that are made up of over 40 different proteins called nucleoporins (Nups). Although diffusion of molecules <55 kDa can occur, most transport through the NPC is mediated by members of the importin superfamily, which recognize nuclear localization sequences (NLSs) or nuclear export sequences (NESs) on cargo molecules for transport into and out of the nucleus, respectively (Ghildyal et al., 2005, 2009; Cardarelli et al., 2008; Cohen et al., 2011). NLSs are usually short stretch(es) of basic residues (monopartite NLS, e.g., that of the simian virus 40 T antigen) which may be separated by 10–15 amino acids (bipartite NLS, e.g., that of nucleophosmin), while the most well characterized NES is the Leucine-rich motif recognized by the exportin CRM-1 (e.g., the NES in HIV Rev protein). Importins function by binding NLSs and docking transiently at various “FG” (Phenylalanine–Glycine repeat containing) Nups within the NPC to effect translocation through it, followed by release within the nucleus facilitated by the guanine nucleotide binding protein Ran (Gorlich and Kutay, 1999). The best studied nuclear import pathway is mediated by the importin-α/β1 heterodimer, where importin-α recognizes and directly binds to the NLSs of the cargo, and importin-β1 mediates binding of the import complex to Nups (Macara, 2001). However, nuclear import can be effected by direct action of importin-β1 or homologs thereof, without a requirement for importin-α (Fulcher and Jans, 2011). In all cases, release within the nucleus occurs through binding of Ran, in its guanosine triphosphate (GTP)-bound form, to importin-β1, or homologs, to effect dissociation of the import complex. Nuclear export is analogous to nuclear import, wherein cargo molecules containing NESs bind importin-β homologs, such as CRM-1, in complex with Ran in its GTP bound state and are transported out of the nucleus; release in the cytoplasm is facilitated by Ran hydrolysis of GTP to guanosine diphosphate (GDP), which leads to dissociation of the export complex (Macara, 2001).

Localization to the nucleolus is less well defined, but is most likely regulated by cargoes interacting with nucleolar residents, possibly the so-called hub proteins (Emmott and Hiscox, 2009). Nucleolar localization signals (NoLSs), similar to NLSs, are short stretches of basic proteins, however, there is a high level of variability in the NoLSs described to date (de Melo et al., 2013). NoLSs are not capable of directing cargo into the nucleus, although very often they overlap the NLS sequences. Interestingly, CRM-1 has been shown to play an important role in nucleolar localization of small nuclear and nucleolar RNA molecules via complex binding mechanisms that lead to masking/unmasking of the NoLS (Verheggen and Bertrand, 2012).

### Coronaviridae and Arteriviridae

Viruses in the *Coronaviridae* family possess a positive (+) ssRNA genome and cluster into two subfamilies including *Coronavirinae* which includes the human coronaviruses, and six genera. The viruses of *Arteriviridae* family are also positive (+) ssRNA viruses and grouped into one genus, Arterivirus.

**FIGURE 2:**
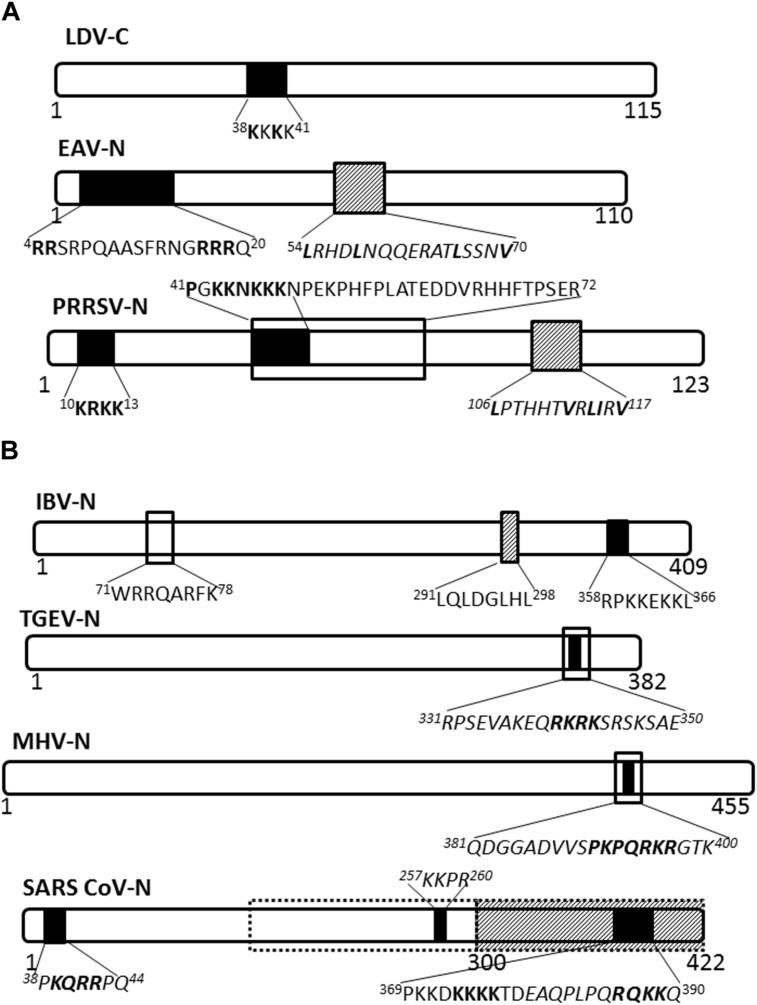
**Nuclear/nucleolar transport signals in nucleocapsid proteins of *Arteriviridae* and *Coronaviridae***. The nucleocapsid proteins of representatives of named virus families are shown schematically. Numbers under the schematics indicate position of amino acid residues, nuclear localization signals (NLSs) are indicated by solid black filled boxes, nuclear export signals (NESs) are indicated by hatched boxes and nucleolus localization/retention signals (NoLS/RS) are indicated by black open boxes. The sequence of the transport signals is shown in single letter format, with essential residues in bold; sequences in italics are predicted but not yet confirmed. **(A)** *Arteriviridae*: LDV, lactate dehydrogenase virus; EAV, equine arteritis virus; PRRSV, porcine respiratory and reproductive syndrome virus. **(B)** *Coronaviridae:* Broken black line indicates predicted NoLS/RS. IBV, infectious bronchitis virus; TGEV, transmissible gastroenteritis virus; MHV, mouse hepatitis virus; SARS-CoV, severe acute respiratory syndrome coronavirus.

The nucleocapsid proteins of viruses belonging to genus Arterivirus localize to the nucleus, as has been shown for lactate dehydrogenase-elevating virus (LDV; Mohammadi et al., 2009), equine arteritis virus (EAV; Tijms et al., 2002) and PRRSV (Rowland et al., 1999). Nuclear import of the LDV nucleocapsid protein is facilitated by a monopartite NLS motif, ^38^KKKK^41^ (**Figure 2A**), probably mediated by the importin-α/β complex (Mohammadi et al., 2009), and whilst the EAV nuclear import has not been clearly defined, nuclear export is facilitated by CRM-1 (Tijms et al., 2002). PRRSV nucleocapsid protein possesses NLS, nucleolar retention/localization signal (NoRS/-LS), and NES motifs that facilitate independent nucleocytoplasmic transport and accumulation in the nucleoli of infected cells (Rowland et al., 1999; Yoo et al., 2003). The NLS motifs that are located in amino acid residues 10–13 (NLS1) and 41–47 (NLS2) facilitate nuclear import via interaction with importin-α/β, while the NES motif is located in amino acid residues 106–117 and works with CRM-1 to facilitate nuclear export (Pei et al., 2008). Disruption of nuclear/nucleolar localization of PRRSV nucleocapsid protein has been shown not to affect the capability to produce infectious particles in MARC-145 cells (monkey kidney cells permissive to PRRSV infection), however, it did result in attenuated viral replication and induced a higher titer of neutralizing antibody in pigs. This suggests the nucleocytoplasmic localization of PRRSV nucleocapsid protein has an important role in pathogenesis of PRRSV infection (Lee et al., 2006; Pei et al., 2008).

Nucleocapsid proteins of coronaviruses IBV and MHV localize to the nucleolus of infected cells facilitated by NLSs, NoRS/-LS, and an NES (Hiscox et al., 2001; Wurm et al., 2001; Chen et al., 2002; Reed et al., 2006; **Figure 2B**). In the nucleolus the nucleocapsid protein may modulate cell division, as evidenced by the fact that cells expressing coronavirus nucleocapsid protein appear to be arrested in the G2/M phase of the cell cycle (Chen et al., 2002; Ding et al., 2014a; McBride et al., 2014). Delay in the cell cycle may promote conditions suitable for virus replication and assembly.

The nucleocapsid protein of IBV has been shown to associate with fibrillarin and nucleolin in the nucleolus (Hiscox et al., 2001). In particular, as also observed for MHV and TGEV nucleocapsid proteins, it causes G2 arrest of the cell cycle that leads to reorganized fibrillarin distribution, where the interaction and/or redistribution are associated with a delay in cell growth (Hiscox et al., 2001; Chen et al., 2002; Ding et al., 2014a).

The nucleolus is a multifunctional nuclear substructure that is central to the normal operations of a cell, with important roles in ribosome synthesis and assembly, cell cycle regulation, transcription regulation, cell senescence and sensing and response to stress (Bartova et al., 2010; Shaw and Brown, 2012; Golomb et al., 2014). The nucleolar proteome consists of more than 500 proteins identified so far; some are long term residents, while others shuttle between the nucleolus, nucleoplasm, cytoplasm, and cell membrane (Andersen et al., 2002; Lam et al., 2005) depending on the metabolic state of the cell. Fibrillarin, nucleolin, and nucleophosmin are major nucleolar proteins that have been studied extensively. Fibrillarin is involved in many post-transcription processes and ribosome assembly; nucleolin serves crucial roles in pre-rRNA processing and may regulate transcription; nucleophosmin is implicated in ribosome assembly, nucleocytoplasmic shuttling and may regulate rDNA transcription [(Hiscox, 2002) and references therein].

The SARS-CoV nucleocapsid protein is predominantly cytoplasmic in infected cells and in cells transfected to express the full length protein. Interestingly, the protein sequence contains three putative NLSs and a putative NES (**Figure 2B**); the latter is non-functional (You et al., 2005, 2007). A bipartite NLS and putative NoLS appear to be functional but are masked in the context of the full length protein by a dominant CRM-1-independent NES in the C-terminus. Additionally, SARS CoV nucleocapsid cytoplasmic localization is facilitated by interaction with 14-3-3 (tyrosine 3-monooxygenase/tryptophan 5-monooxygenase activation protein; Macara, 2001; Surjit et al., 2005). Thus, the SARS-CoV nucleocapsid protein, to the best of our knowledge, does not localize to the nucleus or the nucleolus of infected cells, in contrast to the nucleocapsid proteins of other coronaviruses studied.

### Paramyxoviridae

**FIGURE 3:**
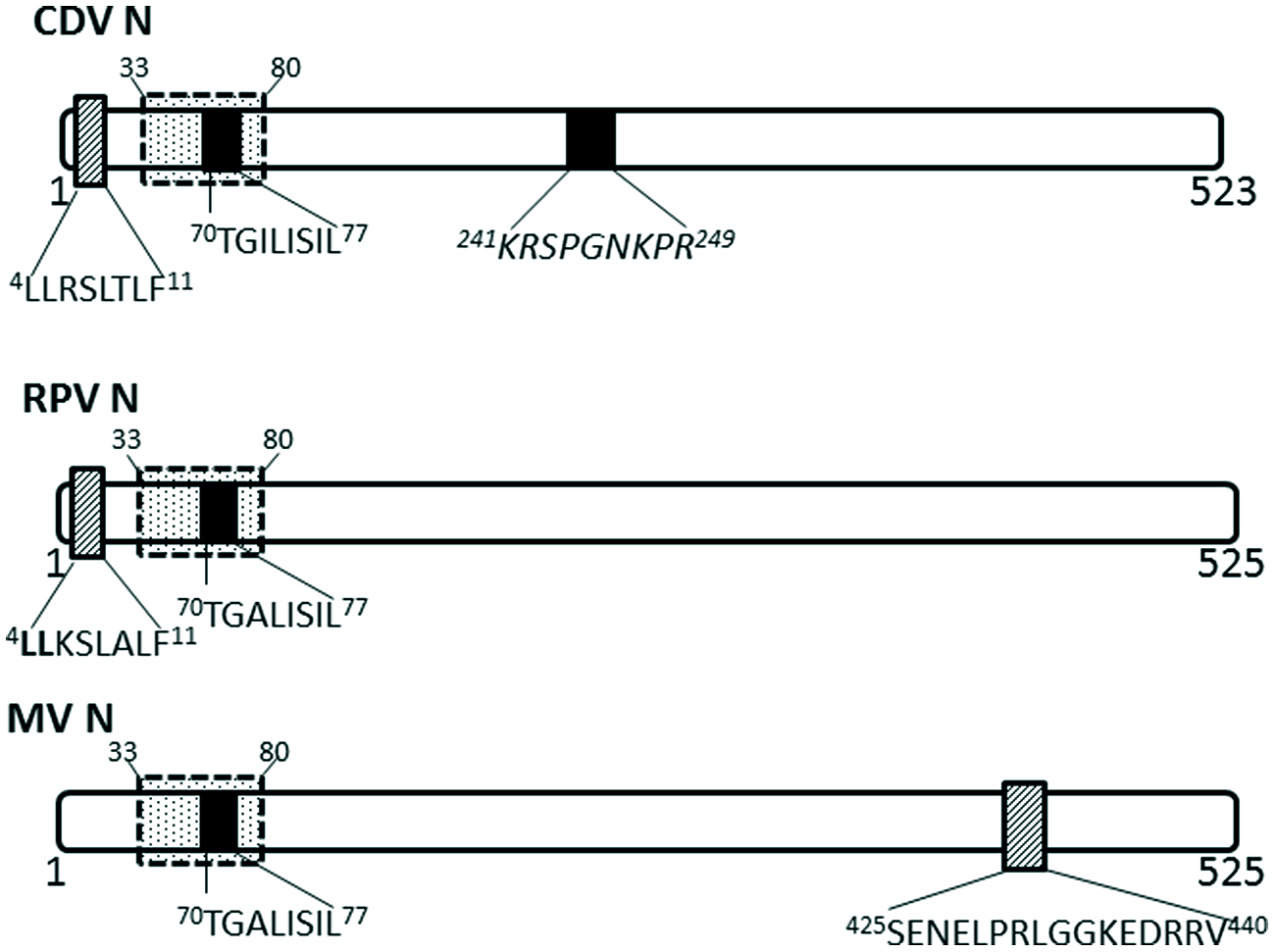
**Nuclear transport signals in nucleocapsid proteins of *Paramyxoviridae***. The NLSs and NESs are indicated as in **Figure 2**; a dashed-line box with dots indicates flanking sequences that have been shown to have a role in nuclear import and retention. CDV, canine distemper virus; RPV, Rinderpest virus; MV, measles virus.

Nucleocapsid proteins of some viruses in the *Paramyxoviridae* family have been shown to undergo transient nuclear localization. The nucleocapsid proteins of canine distemper virus (CDV) and rinderpest virus (RPV) possess conserved NLS and NES motifs; the NES is CRM-1 independent, despite being a Leucine-rich motif (Sato et al., 2006; **Figure 3**). MV infected cells contain nuclear inclusion bodies (composed of N, phosphoprotein and large polymerase; Robbins and Eagle, 1985). More importantly, the nuclear translocation of MV nucleocapsid protein also affects the host immune response by inhibiting the nuclear translocation of STATs, causing disruption of the IFN-α/β and IFN-γ signaling pathways (Takayama et al., 2012). The NLS motif is contained within ^70^TGILISILSLF^80^; while the NES is contained within residues ^425^SENELPRLGGKEDRRV^440^ (Sato et al., 2006).

### Flaviviridae

**FIGURE 4:**
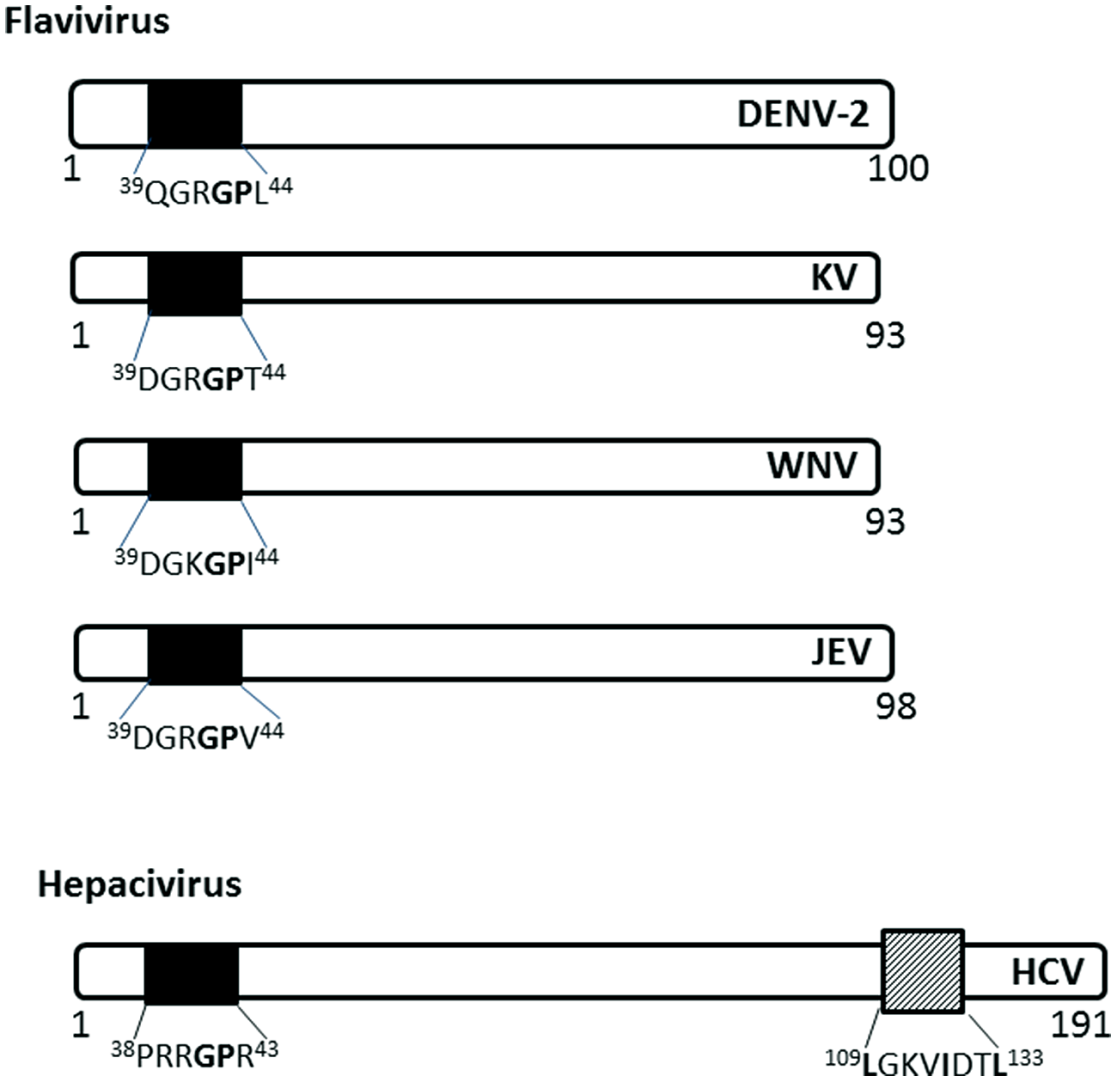
**Nuclear localization signals in C (capsid or core) proteins of *Flaviviridae***. The NLSs for each viral protein and the NES for HCV are indicated as in **Figure 2**. DENV-2, Dengue virus serotype 2, KV, Kunjin virus; WNV, West Nile virus; JEV, Japanese encephalitis virus; HCV, hepatitis C virus.

The capsid (C) protein of several members of the *Flaviviridae* family localize to the nucleus of infected cells using NLSs (**Figure 4**). The Flavivirus C protein is small enough to diffuse across the NE; however, studies have shown that it utilizes active nuclear transport pathways to localize to the nucleus.

The C protein of Dengue virus localizes to the nucleus of infected mammalian cells, starting at 6 h post-infection (h.p.i) and remains at high levels throughout the course of infection (up to 72 h.p.i; Sangiambut et al., 2008). The C protein does not localize to the nucleus of mosquito cells, possibly due to the different availability of carrier proteins or the cellular components involved in nuclear translocation.

The C (core) protein of the Japanese encephalitis virus (JEV) has been shown to localize into the nucleus and associate with the phosphoprotein B23 in the nucleolus (Tsuda et al., 2006). The nuclear localization is required for successful replication in mammalian cell lines and is determined by amino acid residues ^42^GP^43^ of the NLS, with G^42^ being the major determinant (Mori et al., 2005). ^42^GP^43^substitution into A^42-43^ eliminates the nuclear localization ability in both mosquito and mammalian cell lines and decreases infectious virus production in mammalian cell lines and in infected mice. The GP motif is conserved in Dengue, Kunjin, and Hepatitis C virus C proteins (Mori et al., 2005).

Hepatitis C virus C protein has been found in the nucleus in the C-terminally truncated form, particularly in hepatocytes isolated from chronic HCV patients (Falcon et al., 2003), suggesting a possible role in persistence of HCV infection, which often leads to hepatocellular carcinoma. The nuclear localization of HCV C is dependent on importin-α, which recognizes NLS motifs contained within the residues 5–13 (PKPQRKTKR), 38–43 (PRRGPR), and 58–71 (PRGRRQPIPKARRP; Suzuki et al., 2005). HCV C also has a functional CRM-1 dependent NES in residues 109–133 (Cerutti et al., 2011). The nuclear localization has been observed early in infection (~ from 6 h.p.i), similar to that of Dengue C protein, and disruption of the nucleocytoplasmic translocation process reduces the production of infectious particles (Cerutti et al., 2011).

## Nuclear Functions of the Nucleocapsid Proteins of RNA Viruses

Based on current literature and our understanding of nucleocytoplasmic trafficking in the context of virus infection, it can be proposed that the nuclear accumulation of nucleocapsid proteins may serve two functions. Nucleocapsid proteins may have non-structural roles in modulation of nuclear processes in order to maximize virus replication or to inhibit IFN induction and signaling pathways. Alternatively, nucleocapsid proteins may localize coincidentally to the nucleus/nucleolus due to molecular mimicry.

### Virus Replication

The nuclear/nucleolar translocation of the nucleocapsid protein is probably most often associated with optimal virus replication. For example, IBV nucleocapsid redistributes fibrillarin and delays cytokinesis to divert biosynthetic resources from the dividing nucleus to the cytoplasm, where viral replication takes place (Wurm et al., 2001; Chen et al., 2002). The resultant cell cycle arrest/delay would be conducive to higher virus replication through both redirection of biosynthetic machinery for viral replication and delay in cell division. A similar cell cycle arrest has also been observed in other coronaviruses and may be a common consequence of the nucleolar localization of the nucleocapsid protein (Wurm et al., 2001). In nature, these viruses infect terminally differentiated epithelial cells which do not actively divide; it is unclear how the cell cycle modulation observed in cell culture would be useful in a natural infection. The nuclear/nucleolar localization of JEV C protein is also required for optimal virus replication in mammalian cells (Mori et al., 2005). The importance of the nuclear functions of the core protein in flavivirus infection is demonstrated by the inability of some flaviviruses to replicate in enucleated cells (Kos et al., 1975; Lad et al., 1993). Both the Dengue and HCV C protein interact with heterogeneous nuclear RNP K, possibly to regulate host cell transcription, thereby freeing cellular machinery for viral RNA synthesis (Hsieh et al., 1998; Chang et al., 2001).

### Interferon Antagonism

Nucleocapsid proteins of some RNA viruses function in the nucleus to inhibit the potent antiviral IFN response. The innate antiviral response, mediated principally by the action of Type-I IFNs, is one of the earliest responses of the host to viral infection (Conzelmann, 2005). Virus specific intracellular pathogen associated molecular patterns (e.g., viral RNA or dsRNA) are recognized by cellular helicases retinoic-acid-inducible protein I and melanoma-differentiation-associated gene 5 (See and Wark, 2008) initiating a cascade of events resulting in the activation of transcription factors including IFN response factor 3 (IRF3), nuclear factor (NF) κB and activating protein 1 that are subsequently transported into the nucleus to activate transcription of IFN-β. IFN-β mRNA is exported out of the nucleus, where it is translated into protein which is secreted from the cell to induce a secondary cellular response in an autocrine and paracrine manner, by binding to the IFN-α/β receptor. This in turn leads to activation of a second cascade of events involving several effectors and transcription factors such as the STAT proteins. These proteins translocate into the nucleus, interact with IFN-sensitive response elements and activate transcription of a broad range of IFN stimulated genes (ISG). The ISG products mount a concerted immune response which prevents virus replication (See and Wark, 2008).

As mentioned already, MV nucleocapsid protein inhibits the nuclear translocation of STATs, causing disruption of the IFN-α/β and IFN-γ signaling pathways (Takayama et al., 2012). Coronavirus nucleocapsid protein has been shown to have potent IFN antagonistic activity; however, the mechanism varies in different virus species. The MHV nucleocapsid protein interferes with the IFN induced 2′-5′ oligoadenylate synthetase RNase L pathway and can functionally replace the IFN antagonist activity of E3L protein in a recombinant vaccinia virus (Ye et al., 2007), thus contributing to the IFN resistance exhibited by MHV (Rose and Weiss, 2009). The SARS-CoV nucleocapsid protein is also a potent IFN antagonist, however, it is mostly cytoplasmic (discussed above) and inhibits IFN production (Kopecky-Bromberg et al., 2007) rather than IFN signaling. Similar to SARS-CoV, the porcine epidemic diarrhea virus nucleocapsid protein inhibits IFN production via direct interaction with TRAF-associated NF-kB activator binding kinase (TBK1), thus interfering with the TBK1 mediated phosphorylation of IRF3, required for IRF3 dimerization and subsequent nuclear localization, which is critical to IFN activation (Zhao, 2013; Ding et al., 2014b). Nucleocapsid protein’s IFN antagonist activity may not always be dependent on its nuclear localization.

### Molecular Mimicry

The nucleocapsid protein generally has Lysine (K) or Arginine (R) rich RNA binding domains (to associate with viral genome), which resemble nuclear transport signals and these motifs may serve the dual purpose of mediating nuclear localization of nucleocapsid protein coincidental to their main function. This is the basis for molecular mimicry, wherein the K/R-rich RNA binding domains resembling NLS/NoLS motifs, allow the nucleocapsid protein to be transported into the nucleus by cellular nucleocytoplasmic trafficking machinery (Rowland and Yoo, 2003). Other possibilities include association with ribosomal proteins, and passive diffusion. Association with ribosomal proteins in the cytoplasm might result in nucleocapsid protein being coincidentally translocated into the nucleolus, where the ribosomal proteins associate with rRNA to form ribosomal subunits (Tijms et al., 2002). Although termed “coincidental,” nuclear localization of nucleocapsid protein might serve a specific viral purpose by maintaining the nucleocapsid protein:ribonucleotides stoichiometry in the cytoplasm. This is essential in order to produce functional genomes, such that transient nuclear/nucleolar localization of excess nucleocapsid protein can be a means to keep the appropriate concentration of nucleocapsid protein molecules in the cytoplasm (Tijms et al., 2002).

## Potential for Future Therapeutic Strategies

As discussed above, N/NC/C proteins of cytoplasmic RNA viruses have an important nuclear role in addition to their essential cytoplasmic role in virus assembly. This presents an opportunity for a broad ranging antiviral that may inhibit nuclear transport of these proteins, thus indirectly reducing virus assembly, leading to reduced infectious virus production and hence reduced disease. New generations of specific inhibitors of nuclear export (SINE) are well characterized compounds that have low toxicity and good bioavailability and are currently in clinical trials for various cancers and could potentially be re-purposed for virus infections (Azmi et al., 2013a,b; Gao et al., 2014). However, targeting host–pathogen interface is preferable to targeting of cellular processes. Elucidation of the NLS/NoLS motifs of the nucleocapsid proteins and their mechanism of localization should identify interactions that may be modeled for development of small molecule inhibitors (Lundberg et al., 2013); indeed, given the vast array of small molecule inhibitors currently available, it may be possible to identify inhibitors via high throughput screening assays (Wagstaff et al., 2011). Ivermectin and Mifepristone are two such compounds shown to reduce infectious virus titers of several viruses that exploit host nuclear transport machinery (Tay et al., 2013). Determination of the NLS/NoLS also presents an opportunity for development of attenuated vaccine candidates. Specific mutations that disable the nuclear/nucleolar localization function, without hampering the cytoplasmic function of nucleocapsid proteins, should result in viruses that replicate, albeit to lesser degree due to reduced ability to inhibit antiviral responses; mutating the NLS of RSV M protein leads to reduced replication fitness, supporting the viability of such an approach (Ghildyal et al., 2009). That targeting the nucleocapsid protein for attenuation is a viable strategy has been demonstrated for VSV (Sato et al., 2011).

## Summary

Structural proteins of several enveloped RNA viruses localize into the nucleus and/or nucleolus at certain times of the viral life cycle, primarily early in infection. Their nucleocytoplasmic transport is facilitated by nuclear localization/export signals in association with cellular transport proteins. The nucleocapsid protein is a major structural protein of enveloped RNA viruses which serves a structural function in virus assembly. Its function includes protecting the genome against ribonucleases, regulating the fidelity of the replication template, forming the replication complex, and packaging of the genomic RNA for assembly, budding, and particle release. The nucleocapsid proteins of several viruses belonging to *Coronaviridae, Arteriviridae, Flaviviridae*, and *Paramyxoviridae* possess NLS/NoLS and transiently localize into the nucleus/nucleolus during the viral life cycle. In some cases the nuclear localization appears to support viral proliferation by interfering with the host’s immune response or other mechanisms including maintaining the balance of nucleocapsid protein:RNA molecules for optimum production of the functional viral genome. As such, the nuclear localization of nucleocapsid protein of enveloped RNA viruses has potential as a target for the development of live-attenuated vaccines or antiviral drugs, as inhibition of its nuclear localization could negatively impact on the production of infectious viral particles and cell-to-cell spread.

## Conflict of Interest Statement

The authors declare that the research was conducted in the absence of any commercial or financial relationships that could be construed as a potential conflict of interest.
